# Supplementary material for: Ir56d-dependent fatty acid responses in Drosophila uncover taste discrimination between different classes of fatty acids
Source: eLife. 2021 May 5;10:e67878. doi: 10.7554/eLife.67878 (PMC8169106; doi:10.7554/eLife.67878)
Supplement: Transparent reporting form [file elife-67878-transrepform.docx]

***eLife’s* transparent reporting form**

We encourage authors to provide detailed information *within their submission* to facilitate the interpretation and replication of experiments. Authors can upload supporting documentation to indicate the use of appropriate reporting guidelines for health-related research (see [EQUATOR Network](http://www.equator-network.org/%20)), life science research (see the [BioSharing Information Resource](https://biosharing.org/)), or the [ARRIVE guidelines](http://www.plosbiology.org/article/info:doi/10.1371/journal.pbio.1000412) for reporting work involving animal research. Where applicable, authors should refer to any relevant reporting standards documents in this form.

If you have any questions, please consult our Journal Policies and/or contact us: [editorial@elifesciences.org](mailto:editorial@elifesciences.org).

**Sample-size estimation**

- You should state whether an appropriate sample size was computed when the study was being designed
- You should state the statistical method of sample size computation and any required assumptions
- If no explicit power analysis was used, you should describe how you decided what sample (replicate) size (number) to use

Please outline where this information can be found within the submission (e.g., sections or figure legends), or explain why this information doesn’t apply to your submission:

Sample sizes were estimated based on previous literature. We state in the methods section: “Sample sizes for behavioral and functional imaging experiments are consistent with previous studies (Kirkhart & Scott, 2015; Tauber et al., 2017). Generally, ~30 flies were used for each experimental or control group for behavioral experiments and ~10-12 flies per group for imaging experiments.”

**Replicates**

- You should report how often each experiment was performed
- You should include a definition of biological versus technical replication
- The data obtained should be provided and sufficient information should be provided to indicate the number of independent biological and/or technical replicates
- If you encountered any outliers, you should describe how these were handled
- Criteria for exclusion/inclusion of data should be clearly stated
- High-throughput sequence data should be uploaded before submission, with a private link for reviewers provided (these are available from both GEO and ArrayExpress)

Please outline where this information can be found within the submission (e.g., sections or figure legends), or explain why this information doesn’t apply to your submission:

Behavioral and functional imaging experiments were performed ~3 times per week. We state in the methods: “Experiments were run blinded, ~3 times per week until completion.”. Each organism represents a biological replicate, and the assay itself has technical replicates built in for each animal (e.g. three presentations of each tastant). There were no outliers that were excluded.

**Statistical reporting**

- Statistical analysis methods should be described and justified
- Raw data should be presented in figures whenever informative to do so (typically when N per group is less than 10)
- For each experiment, you should identify the statistical tests used, exact values of N, definitions of center, methods of multiple test correction, and dispersion and precision measures (e.g., mean, median, SD, SEM, confidence intervals; and, for the major substantive results, a measure of effect size (e.g., Pearson's r, Cohen's d)
- Report exact p-values wherever possible alongside the summary statistics and 95% confidence intervals. These should be reported for all key questions and not only when the p-value is less than 0.05.

Please outline where this information can be found within the submission (e.g., sections or figure legends), or explain why this information doesn’t apply to your submission:

In each figure legend, we indicate the statistical analysis used, the sample size, the corresponding test for multiple comparisons, and the exact *P*-value of the statistical analysis. In the methods section, we justify the statistical analyses used. We state: “Measurements of PER and aversive taste memory were not normally distributed and so the non-parametric Kruskal-Wallis test was used to compare two or more genotypes. To compare two or more genotypes and two treatments, a restricted maximum likelihood (REML) estimation was used. For data that was normally distributed (calcium imaging data), a one-way or two-way analysis of variance (ANOVA) was used for comparisons between two or more genotypes and one treatment or two or more genotypes and multiple treatments, respectively. All post hoc analyses were performed using Sidak’s multiple comparisons test. Statistical analyses and data presentation were performed using InStat software (GraphPad Software 8.0; San Diego, CA).”

(For large datasets, or papers with a very large number of statistical tests, you may upload a single table file with tests, Ns, etc., with reference to sections in the manuscript.)

**Group allocation**

- Indicate how samples were allocated into experimental groups (in the case of clinical studies, please specify allocation to treatment method); if randomization was used, please also state if restricted randomization was applied
- Indicate if masking was used during group allocation, data collection and/or data analysis

Please outline where this information can be found within the submission (e.g., sections or figure legends), or explain why this information doesn’t apply to your submission:

Masking was used for group allocation during all behavioral experiments. We state in the methods: “Experiments were run blinded, ~3 times per week until completion.”

**Additional data files (“source data”)**

- We encourage you to upload relevant additional data files, such as numerical data that are represented as a graph in a figure, or as a summary table
- Where provided, these should be in the most useful format, and they can be uploaded as “Source data” files linked to a main figure or table
- Include model definition files including the full list of parameters used
- Include code used for data analysis (e.g., R, MatLab)
- Avoid stating that data files are “available upon request”

Please indicate the figures or tables for which source data files have been provided:

We have included source data files with our submission. This file includes all the raw data associated with each figure of the manuscript.
